# Causes and Treatment of Dental Caries

**Published:** 1887-12

**Authors:** Geo. P. Rishel

**Affiliations:** Hornellsville, N. Y.


### CAUSES AND TREATMENT OF DENTAL CARIES.

BY GEO. P. RISHEL, D. D. S., HORNELLSVILLE, N. Y.

READ BEFORE THE JOINT MEETING OF THE SIXTH, SEVENTH AND EIGHTH DISTRICT DENTAL SOCIETIES AT BUFFALO, OCTOBER, 1887.

Vitality is a force resulting from harmonious functional activity. In a state of vigorous health there is not only a correspondence between waste and repair, but the vital energy may almost be measured by the rapidity of these physiological changes. Supply is not to be measured by the amount of food taken, but by the amount utilized in tissue building. Assimilation may depend, not only upon the general health, but upon such influences as climate, temperament, habit, and even the emotions. The splendid teeth of the hardy Scotchman are not, as popularly supposed, the result of an oat-meal diet, but are due to his environments, and to constitutional and temperamental attributes which enable him to assimilate the food taken. Teeth below medium in structure are the result of imperfect or non-assimilation of the materials so abundantly furnished, and to be found upon every table. It is not contended that tooth-structure may not be improved by a diet which contains more than the average amount of lime-salts. Our present knowledge of the laws which affect nutrition is too limited, and the clinical evidence in support of this theory too universal, to admit of doubt; it is, nevertheless, the opinion of the writer, that the food habit is one of the least of the many influences affecting the dental organs.

Magitot having constructed a map of various shades, for the purpose of showing the relative frequency of caries in the eighty-six military departments of France, during the period from 1837 to 1849 inclusive (13 years), and based upon exemption for loss of teeth, after discussing at length the influences of climate, altitude, food, etc., arrives at the following conclusion: We have already remarked upon the influence of race in the question which occupies us, and it seems evident that we must have recourse to considerations of *this nature* to explain the various tints of the chart.

Next in importance, and to a certain extent growing out of the peculiarities of race-development, is that of temperament. Under its influence the trifacial—the nutrient nerve of the teeth—performs its functions. Under its influence the different varieties of tooth-structure are formed, and by reason of its influence may be seen the various conditions usually described as hard and soft decay, or black, yellow and white caries, and generally supposed to be a result of effects produced by different kinds of acids.

There seems to be considerable evidence that vital energy, whether displayed in a general way or made manifest by the ability of special organs to resist the encroachment of disease, is largely dependent upon and influenced by temperament. Time and space will not permit a thorough discussion of this phase of the subject, and I will simply add a quotation from an article written by me and published in the July number of the *Dental Cosmos* for 1882.

"Mal-nutrition, therefore, we consider to be the principal cause of defective tooth-structure. Depressing systemic influences, resulting from nervous irritability, and produced by high living, over-stimulation, and the rush for wealth, place and power, are destroying not only the teeth, but the lives of the present generation. Even the children are not exempt, and from the time they are large enough to 'show off' at the competitive examinations of school-days, and the cramming process of college life, everything is on the high-pressure principle."

Patients of the nervous or lymphatic temperament suffer first and most, owing to the quick response to every influence of the one, and the slow but unresisting qualities of the other.

The stomach, when deprived of its vitality may be quickly dissolved by the gastric juice, which, during life, made no impression upon it, and in like manner the teeth, when deprived of even a portion of vital energy, become an easy prey to the hungry assailants constantly lurking within the oral cavity.

It is well known that during periods of sickness of a special debilitating character, those portions of the body possessing a low grade of vitality, as the teeth and hair, suffer most, and but very slowly partake of the reparative process, and not until the general system is far advanced in the way of improvement.

Caries is, therefore, primarily and to a large extent the result of imperfect assimilation, with its concomitants, lowered vitality and defective tooth-structure, modified and controlled by nervous influences, and last, but not least, to chemical disintegration, modified and aided by micro-organisms, some of which have the power to produce acid fermentation.

Dr. W. D. Miller, of Berlin, who was the first to produce artificial caries with all the essential characteristics found in the mouth, and to whom we are indebted for much light upon this subject, says : "The first stage of caries of the teeth, *i.e.*, the extraction of the lime-salts, is, for the most part, caused by acids which are generated in the mouth by fermentation. Decalcification of the enamel signifies total destruction of that tissue; of the dentine there remains, after decalcification, a tough, spongy mass, which becomes subject to the invasion of enormous numbers of fungi (leptothrix threads, bacilli, micrococci, etc)."

The fungi produce anatomical and pathological changes in the deep layers, stop up the canaliculi, and necessarily lead, sooner or later, to the death of the dentinal fibrils. The outer layers of dentine, thereby deprived of nourishment, die, and fall a prey to putrefactive agents.

We may accordingly look upon caries of the teeth as consisting of three stages: (1) decalcification, (2) infection and devitalization of the decalcified dentine, (3) putrefaction of the devitalized dentine, though it would not be easy to say just where one stage ceases and the other begins.

It must be evident to the most superficial observer that the foundation of all treatment rests upon diagnosis, and that in proportion as we have accurate information in regard to the causes which, directly or indirectly, lead to the disease known as dental caries, are we, as a profession, competent to render valuable services to our patients.

The influences of race, temperament and habits of life, are for the most part beyond our control, but the *knowledge* of these influences will often aid us in deciding as to the best mode of treatment. That general condition of the dental organs, including loss of tooth-structure, frail walls and a tendency to inflammatory conditions, which in a patient of the nervo-lymphatic temperament suggests to the intelligent operator plastic fillings and the employment of the most careful means for lessening the effects of thermal changes, makes no such appeal when presented to us under similar circumstances, but by a patient of the bilio-sanguine temperament. In like manner that grade of pulpitis, which in the one instance may be readily pronounced incurable and calls for immediate extirpation, may in the other be just as surely restored to life-long usefulness, and presents but few if any difficulties.

The first step in the proper treatment of dental caries consists of such persistent and intelligent use of flòss silk, tooth powder and brush, as will secure comparative cleanliness, and of the daily employment of a mouth-wash which will secure a more or less complete sterilization of the oral cavity. Listerine will meet many, if not all, of the requirements, and may be used full strength or diluted. The formula for a valuable and much cheaper, though not so pleasant a mouth-wash, recommended by Dr. Miller and published in the September number of the INDEPENDENT PRACTITIONER, is as follows:

| | | |
|---|---|---|
| ℞ | Thymol, | gr. iv. |
| | Acid benzoic, | gr. xxxxv. |
| | Tr. eucalyptus, | ʒ iii ss. |
| | Aqua, | Fl. ℥ xxv. |

Notwithstanding the great improvement in appliances, and the corresponding advance made in the mere mechanical treatment of caries, the importance of preliminary medicinal treatment must not be ignored. The first steps in the way of loeal treatment consist in the application of the rubber dam, the cutting away of frail and ragged margins, and the removal of such portions of decalcified dentine as can be successfully accomplished without pain. If sensitive, make an application of Naboli No. 2, allowing it to remain about five minutes, wipe out the cavity and apply No. 3 for six or seven minutes, and remove all traces of No. 3 by using No. 4, absolute alcohol, or chloroform. After using the hot air syringe it will be found that the final work of preparing the cavity causes but little if any pain. It should now be saturated with carbolic acid, dried, and again saturated with a solution of copal in ether, the margins polished, and the filling inserted.

The employment of Naboli as a pain obtunder is based upon scientific principles, and failure can only be due to improper methods. No. 3 is composed of glycerine, tannic acid and a minute quantity of chloral.

The irritation caused by the great affinity of glycerine for water, and the astringent properties of tannic acid (the combined influence of which extracts moisture and produces contraction of the dentinal fibrils), is modified and controlled by the chloral. The power of the nerve filaments to transmit sensation is still further reduced by the application of absolute alcohol and hot air. The carbolic acid destroys the micro-organisms to be found in every cavity, and stimulates the pulp to increased efforts in its resistance to the encroachment of disease.

By reason of the dry and contracted condition of the dentinal fibrils, the copal varnish enters deeply into, and hermetically seals the ends of the dental tubuli, secures for the pulp comparative immunity from the injurious effects of thermal changes, and for the filling protection from pulp exudations.

